# Exploring early life social and executive function development in infants and risk for autism: a prospective cohort study protocol of NICU graduates and infants at risk for cerebral palsy

**DOI:** 10.1186/s12888-024-05779-z

**Published:** 2024-05-14

**Authors:** Kelsie A. Boulton, Dabin Lee, Ingrid Honan, Natalie L. Phillips, Catherine Morgan, Cathryn Crowle, Iona Novak, Nadia Badawi, Adam J. Guastella

**Affiliations:** 1 Clinic for Autism and Neurodevelopmental (CAN) research, Brain and Mind Centre, Children’s Hospital Westmead Clinical School, Faculty of Medicine and Health, University of Sydney, Sydney, Australia; 2 Child Neurodevelopment and Mental Health Team, Brain and Mind Centre, University of Sydney, Sydney, Australia; 3 Cerebral Palsy Alliance Institute, Discipline of Child and Adolescent Health, The University of Sydney, Sydney, Australia; 4 Grace Centre for Newborn Intensive Care, Sydney Children’s Hospital Network, Sydney, Australia

**Keywords:** Autism, NICU, Cerebral palsy, Early markers, Social delay, Executive function delay

## Abstract

**Background:**

Delays in early social and executive function are predictive of later developmental delays and eventual neurodevelopmental diagnoses. There is limited research examining such markers in the first year of life. High-risk infant groups commonly present with a range of neurodevelopmental challenges, including social and executive function delays, and show higher rates of autism diagnoses later in life. For example, it has been estimated that up to 30% of infants diagnosed with cerebral palsy (CP) will go on to be diagnosed with autism later in life.

**Methods:**

This article presents a protocol of a prospective longitudinal study. The primary aim of this study is to identify early life markers of delay in social and executive function in high-risk infants at the earliest point in time, and to explore how these markers may relate to the increased risk for social and executive delay, and risk of autism, later in life. High-risk infants will include Neonatal Intensive Care Unit (NICU) graduates, who are most commonly admitted for premature birth and/or cardiovascular problems. In addition, we will include infants with, or at risk for, CP. This prospective study will recruit 100 high-risk infants at the age of 3–12 months old and will track social and executive function across the first 2 years of their life, when infants are 3–7, 8–12, 18 and 24 months old. A multi-modal approach will be adopted by tracking the early development of social and executive function using behavioural, neurobiological, and caregiver-reported everyday functioning markers. Data will be analysed to assess the relationship between the early markers, measured from as early as 3–7 months of age, and the social and executive function as well as the autism outcomes measured at 24 months.

**Discussion:**

This study has the potential to promote the earliest detection and intervention opportunities for social and executive function difficulties as well as risk for autism in NICU graduates and/or infants with, or at risk for, CP. The findings of this study will also expand our understanding of the early emergence of autism across a wider range of at-risk groups.

## Background

Early detection of developmental delays is crucial to provide optimal support. Identifying early markers that may signpost elevated risk for developmental delay is one way to improve these early detection and support efforts. Literature to date has shown that delays in early social and executive function markers may be predictive of later developmental delays and eventual neurodevelopmental diagnoses, such as autism [1, 2]. Two at-risk infant groups that can show significant delays associated with social and executive function and have an elevated risk of meeting criteria for autism spectrum diagnoses include neonates admitted to the Neonatal Intensive Care Unit (NICU) and infants with, or at risk for, cerebral palsy (CP). Factors necessitating NICU admissions, such as premature birth, low birth weight and other medical complications like congenital heart disease, have been linked to developmental delays [3], while up to 95% of individuals with CP present with at least one additional medical, neurological, or neurodevelopmental condition, extending beyond motor impairments core to the CP diagnosis [4].

To illustrate, delays in both social development and executive function have been repeatedly observed across infants admitted to the NICU and infants with CP [5–10]. For instance, cross-sectional studies of these high-risk infants have reported social impairments spanning from social-communicative difficulties in early childhood to social isolation and withdrawal in adolescence and adulthood [6, 9–15]. These delays have been found to confer lifelong challenges including decreased quality of life, relationship issues and fewer educational and vocational opportunities [16–18]. Additionally, there is growing evidence of attention and executive function delays in both NICU graduates and infants with CP, with attentional deficits detected from the first years of life and delays in executive function reported across the lifespan [7, 19–23]. Similar to social impairments, these delays in executive function have been associated with poorer outcomes across academic achievement, employment and quality of life [24, 25], further underscoring the importance of detecting delays early in development and providing appropiate supports.

While social and executive function impairments are common and considerably impact on daily function in and of themselves, these high-risk infants are also at heightened risk for developing autism. An elevated risk for autism has been linked with risk factors that lead to NICU admissions [26, 27], with early medical complications like low birth weight, congenital heart disease and other birth defects being associated with an increased incidence of autism [28–31]. Further, it is estimated that up to 30% of children with CP may go onto receive an additional diagnosis of autism [32]. Early identification of social and executive function markers in these high-risk infants would thus help us to develop reliable markers of risk for autism and provide supports and interventions as early as possible.

Despite this, the identification of delay in social and executive functioning domains and a later diagnosis of autism is often not made until years after presentation to health services. Early detection and intervention are critical to safeguard the developmental trajectories of social and executive function and to optimize developmental outcomes [33–35]. To date, however, there has been relatively little research exploring the early identification of these delays in high-risk infant cohorts, particularly in the first months of life. While past research has identified early signs of social and cognitive delays in the first year of life in NICU graduates [36–39], there has been a lack of studies exploring early markers that can predict later social and executive function delays and autism diagnoses. Similarly, no research to date has evaluated early predictors of social and executive function delays in infants with CP, with much of the research efforts focused on motor impairments [40]. This paucity of research highlights the need to explore early divergences in social and executive function in these high-risk cohorts during the first months of life, and to identify markers that can predict later developmental delays. Alongside this, despite the high prevalence of autism in both CP and NICU graduates, research examining the early emergence of autism in NICU graduates is scarce [41, 42] and is non-existent for CP. Instead, much of the extant knowledge comes from studies of infants at familial risk for autism [43, 44]. The search for early markers of autism in infants with CP and NICU graduates is therefore critical, both to expand our understanding of early markers of autism, and to ultimately advance our ability to detect and intervene across a broader range of at-risk infants.

While much of the evidence considering risk for autism comes from familial risk studies (e.g., infant siblings of autistic children), it has provided valuable insights into the early emergence of autism and its associated developmental delays. Thus far, reliable markers of autism have been established from the second year of life onwards. These markers include atypical social interaction and communication behaviours as well as distinct profiles of early temperament, motor development and attention [1, 45–47]. However, there has been much less clarity on whether these markers exhibit predictive value in the first year of life, and particularly in the first 6 months [48, 49]. Nevertheless, it is becoming increasingly apparent that the subtle and transient signs of atypical development in the first year *can* be detected by adopting more sensitive measures [50]. For example, studies adopting eye-tracking technology have identified atypical gaze patterns as one promising early marker of later autism [51–55]. Likewise, atypicalities in brain structure and function have demonstrated predictive value from as early as the first few months of life, supporting the speculation that neural measures may provide a window for earlier detection, with neural alterations preceding behavioural changes in autism [1, 50, 56–58]. Moreover, recent research has proposed a link between cortical and epidermal development, suggesting that skin barrier integrity and skin lipid profiles could also represent very early indicators for neurodevelopmental divergence [59, 60]. Taken together, the existing evidence underscores the need to take a multi-modal approach that integrates both behavioural and neurobiological measures when examining developmental delays in early infancy. No previous studies have adopted such an approach to prospectively explore the early emergence of social and executive function impairments and risk for autism in infants with CP and NICU graduates. A prospective investigation of early social and executive function delays in these high-risk groups is therefore warranted, to better understand which infants are at increased risk for later social and executive function impairments and may go onto receive an autism diagnosis.

This article presents the protocol for a prospective longitudinal study, which tracks early social and executive function development as well as the risk for autism in two high-risk cohorts, specifically NICU graduates and infants with, or at risk for, CP. Infants are tracked from 3 to 7 months through to 2 years of age. The study will take a multi-modal approach to track early social and executive function development, using behavioural, neurobiological, and caregiver-reported everyday functioning markers. The data from this study has the potential to: (i) provide benchmarks for the early detection of delays in social and executive function and risk for autism in NICU graduates and infants with CP; and (ii) inform on interactions between social and executive function delays with related domains of broader development (e.g., motor, cognitive, language, caregiver well-being and caregiver perceived stigma). With these data, we will be able to establish prediction models for social and executive function delays as well as risk for autism in these high-risk infants, which will then help inform the development of more targeted early interventions. Finally, this study will provide a broader understanding of the early emergence of autism across a wider range of at-risk infants.

### Study aims

The primary aim of this study is to identify markers of social and executive function delay in high-risk infants from as early as 3–7 months of age, and to explore how these markers may relate to an increased risk for autism at 2 years of age. High-risk infants will include NICU graduates and/or infants with, or at risk for, CP. Given that social and executive function impairments often occur alongside difficulties in motor, cognitive and language development, as well as poor parental wellbeing, the secondary aim is to examine the associations between the early markers of social and executive function and these broader developmental domains.

## Methods

### Study design and setting

In this prospective longitudinal study, initial assessments will be conducted when participants are 3–7 (T1) months old, and follow-up assessments will be administered when they are 8–12 (T2), 18 (T3) and 24 (T4) months old. The assessment sessions will take place at the Brain and Mind Centre, University of Sydney, or the Cerebral Palsy Alliance (CPA) early diagnosis clinics.

### Participants

This longitudinal study will consecutively recruit 100 infants at the age of 3–12 months old, with age corrected for prematurity. Two groups of infants will be recruited for the study: (1) infants who have been admitted to the NICU; and/or (2) infants with, or at risk for, CP. Infants with vision or hearing impairments or severe medical conditions that may interfere with task completion will be excluded. Families will be referred to the study through CPA early diagnosis clinics, NICUs and NICU follow-up clinics. In the interest of maximising recruitment potential, the upper age cut-off for recruitment has been extended from 7 months to 12 months of age. However, recruitment efforts will primarily be focused on infants aged 3–7 months old. Participants aged 8–12 months old at intake will begin assessments at Time 2 and will be followed up at 18 (T3) and 24 (T4) months old. Informed consent to participate will be obtained from caregivers or guardians of infants recruited into the study in line with existing human research ethics approval (2021/HE000937).

### Study procedure

**Fig. 1:**
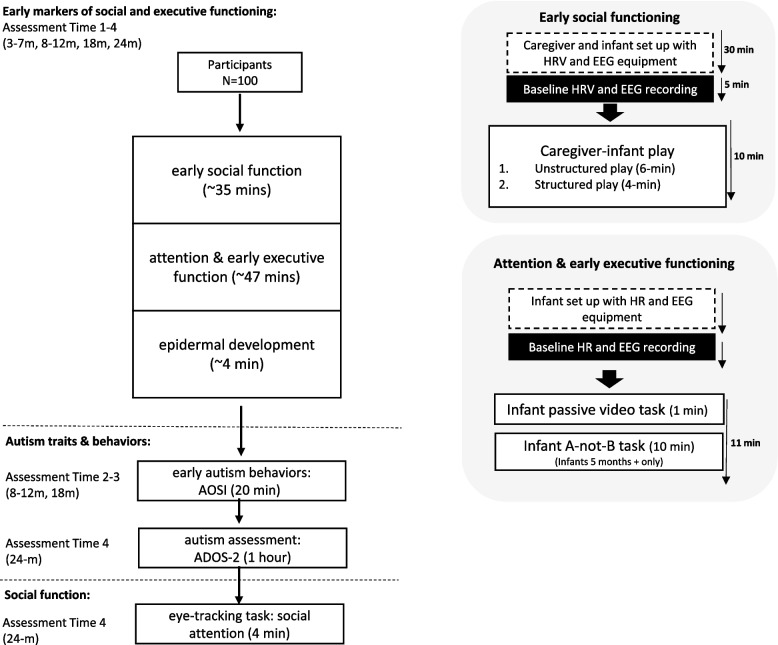
Assessment procedure

The development of social and executive function will be assessed at 3–7 (T1), 8–12 (T2), 18 (T3) and 24 (T4) months, using behavioural (assessed by laboratory-based tasks), neurobiological (brain activity, physiological measures, epidermal development) and caregiver-reported everyday functioning markers. In addition to this, early autistic behaviours will be measured at 8–12 (T2) and 18 (T3) months. The final assessment at 24 months (T4) will also include a comprehensive assessment for autism, the Autism Diagnostic Observation Schedule, 2nd Edition (ADOS-2) and an eye-tracking task to measure social attention. The assessment procedure is outlined in Fig. 1.

### Measures

#### Early social function

Early social function will be measured both behaviourally and neurobiologically, during the completion of social play tasks. Both the caregiver and infant will be set up with an electroencephalogram (EEG) and mindware physiological recording equipment. Caregivers and infants will be fitted with a 24-channel EEG cap (eego™ sports 24, ANT Neuro). The EEG data will be recorded at a sampling rate of 2 kHz using the 24-channel shielded saline-soaked waveguard net and eego™ amplifier, which has four bipolar input channels and two TTL-based trigger inputs, integrated with the eego™ software package. The physiological recording equipment will measure heart rate variability and respiratory rate. Baseline neural and physiological measures will be recorded for 5 minutes while the caregiver and infants watch a Baby Einstein video (© 2002, The Baby Einstein, LLC), which is commonly used for baseline assessments [61, 62]. Infants will then engage in one unstructured play task and two structured play tasks. Caregivers and infants will complete these play tasks while continuing to wear the EEG and physiological recording equipment. All tasks will be recorded for offline behavioural coding and the neurobiological data will be analysed using previously published methods [63–66].

##### Unstructured play

The unstructured play task will involve 6 minutes of free play between the caregiver and infant. Behavioural coding of the unstructured play task will be performed using two coding systems: (1) A quantitative coding system developed by our team based on prior research on caregiver-infant behavioural synchrony [67, 68], where the behaviour of each dyad will be microcoded in 0.01-second frames for important behavioural categories (e.g., eye gaze, social facial expressions, mirroring); (2) The Manchester Assessment of Caregiver-Infant Interaction (MACI [69];), a global rating scale covering broad features of caregiver, infant and dyadic interaction quality. The MACI has been used to capture interaction quality in typically developing infants, as well as those at risk for autism [70].

##### Structured play

**Still face paradigm** The still face paradigm will assess caregiver-infant interaction under exposure to socio-emotional stress [71]. This paradigm occurs in three phases: (1) play: caregiver engages in free play with infant; (2) still face: caregiver stops engaging with infant and maintains a neutral face; (3) reunion: caregiver resumes playing with infant. Behavioural coding of the still face procedure will be performed using the infant and caregiver engagement phases (ICEP) coding system [72, 73], which microanalyses the caregiver and infant’s behavioural responses during the still face paradigm.

**Imitation task** The imitation task will assess facial mimicry. This task will involve the assessor demonstrating two target gestures (tongue protrusion and mouth opening) to the infant [74]. Imitation will be measured by computing the degree to which the child’s response matches the assessor’s modelling of the target gesture. Given that neonatal imitation is bound to a temporal window of approximately 0 to 3 months of age, the imitation task will only be administered for infants aged 3–7 months old (T1).

#### Attention and early executive function

Attention and early executive function will also be measured behaviourally and neurobiologically. The infant will continue to wear the EEG cap and the mindware physiological recording equipment for the attention and executive function tasks. From the EEG, frontal lobe functioning will be measured via brain electrical activity (EEG power) and functional connectivity (EEG coherence). The physiological recording equipment will measure heart rate, which will provide a biological measure of attention [75]. Infants will complete all tasks while wearing the recording equipment. All tasks will be recorded for offline behavioural coding and the neurobiological data will be analysed using previously published methods [63, 64].

##### Attention

**Orienting task** Orienting attention will be measured by a modified version of the “Orientation” items of the NICU network neurobehavioural scale (NNNS) assessment [76]. This task involves presenting infants with a series of inanimate visual (ball), auditory (rattle) and visual/auditory stimuli. The infant’s orienting responses will be scored according to the NNNS manual.

**Passive viewing attention task** The passive viewing attention task will involve presenting infants with an engaging and brief 1-minute Sesame Street video, which has been used in previous studies [77, 78]. The peak look duration (i.e., longest look at the video) and shift rate (i.e., number of looks at the video) will provide a measure of sustained and orienting attention, respectively. Heart rate deceleration will provide a physiological measure of sustained attention [75].

##### Early executive functioning

**A-not-B task** The A-not-B task requires infants to observe a toy as it is hidden in one of two locations (A [left of midline], B [right of midline]) and to find the toy after a delay. Once the infant finds the hidden toy on two consecutive trials, the side of hiding will be reversed. A looking version of this task will be employed in consideration of the potential motor impairments of the participants and to minimise the motion artifact in the EEG recording [79]. The reaching and looking versions of the task have been found to produce comparable performance in infants [80].

#### Epidermal development

Epidermal development will be measured by measuring infants’ skin barrier functioning and skin lipid profiles. Skin barrier functioning will be measured non-invasively via transepidermal water loss, using a Vapometer® SWL5 (Delfin Technologies Ltd.), with elevated transepidermal water loss indicating a disrupted epidermal barrier [81]. The Vapometer, similar to a thermometer, will be placed on the skin of the child’s forearm for 5–16 seconds. Skin lipids will also be collected non-invasively by using the D-SQUAME standard adhesive discs, the discs will be applied to the skin and pressed onto the skin for 5–10 seconds before it is removed [82]. Epidermal protein levels will be measured through mass spectrometry.

#### Caregiver-reported everyday function

Caregivers will be asked to complete questionnaires on children’s everyday behaviour, specifically on their everyday social and executive function, and early autism traits and behaviours (see Table 1). The following questionnaires will be administered from Time 1, depending on the age range for which each questionnaire has been validated. At the initial assessment, caregivers will also be asked to provide basic demographic information, including the child’s date of birth, gender, prematurity, and history of medical complications.

**Table 1:** Timeline of caregiver questionnaires

Measures	Administration			
	Time 1 (3-7 m)	Time 2 (8-12 m)	Time 3 (18 m)	Time 4 (24 m)
*Everyday social functioning*				
Ages and Stages Questionnaires, Third Edition (ASQ-3)	x	x	x	x
Ages and Stages Questionnaires: Social-Emotional, Second Edition (ASQ:SE-2)	x	x	x	x
The Infant Behavior Questionnaire-revised short form (IBQ-r SF) / The Early Childhood Behavior Questionnaire short form (ECBQ-SF)	x	x	x	x
Sensory Experiences Questionnaire – short form (SEQ-SF)	x	x	x	x
The Communication and Symbolic Behavior Scales Developmental Profile Infant-Toddler Checklist (CSBS-DP ITC)	x ^a^	x	x	x
The Brief Infant-Toddler Social and Emotional Screening (BITSEA)		x ^a^	x	x
Brief Infant Sleep Questionnaire – Revised (Short Form; BISQ – R SF)	x	x	x	x
*Everyday attention and executive functioning*				
The Early Executive Functions Questionnaire (EEFQ)		x ^a^	x	x
*Early autism behaviour*				
The Baby and Infant Screen for Children with aUtIsm Traits Part 1 (BISCUIT-Part 1)			x	x
*Social and executive functioning, and child wellbeing at 24 months*				
The Vineland Adaptive Behaviour Scales – Third Edition (Vineland-3)				x
The Behavior Rating Inventory of Executive Functioning – Preschool Version (BRIEF-P)				x
The Child Behavior Checklist for Ages 1.5–5 (CBCL)				x

^a^ Only administered if questionnaire has been validated for child’s age

#### Everyday social functioning

*The Ages and Stages Questionnaire, Third Edition (ASQ-3* *[*83*];)* is a caregiver completed questionnaire that provides ratings on early development in infants and children from 1 month to 5.5 years of age [83]. The ASQ-3 assesses development in five areas: communication; gross motor; fine motor; problem solving; personal-social. The ASQ-3 has been found to exhibit good validity and reliability [83], and has also been validated in preterm children [84].

*The Ages and Stages Questionnaire: Social-Emotional, Second Edition (ASQ:SE-2* *[*85*];)* is a caregiver-completed questionnaire that screens emotional and social behaviour in infants and children from 1 to 72 months. It measures self-regulation, compliance, social communication, adaptive functioning, autonomy, affect and interaction with people. The ASQ:SE-2 has been identified as one of the most comprehensive and psychometrically sound measures of early social-emotional development [86], and has been used with preterm infants [87].

*The Communication and Symbolic Behavior Scales Developmental Profile Infant-Toddler Checklist (CSBS-DP ITC* *[*88*];)* is a 24-item caregiver report questionnaire that assesses seven key predictors of later language delays: emotion and use of eye gaze, use of communication, use of gestures, use of sounds, use of words, understanding of words, and use of object. The CSBS-DP ITC is used as a developmental screen for infants aged 6–24 months, and has demonstrated good psychometric properties, with evidence for concurrent validity, test-retest reliability and predictive validity [89].

*The Infant Behavioral Questionnaire-revised Short Form (IBQ-r SF* *[*90*,*^91^*];)* is a parent-report questionnaire that measures general patterns of behaviour and temperament in infants aged 3 to 12 months. *The Early Childhood Behavior Questionnaire – Short Form (ECBQ-SF* *[*92*];)* is a parent-report questionnaire that measures general patterns of behaviour and temperament in infants and toddlers aged 18 to 36 months. Both the IBQ-r SF and the ECBQ-SF have good reliability and validity and have been shown to predict laboratory measures of attention and temperament [91, 93].

*The Brief Infant-Toddler Social and Emotional Screening (BITSEA* *[*94*];)* is a 42-item caregiver completed questionnaire that assesses social-emotional and problems and delays in competence in infants aged 12 to 36 months. The BITSEA provides general Problem and Competence Total scores and has demonstrated excellent psychometric properties [94, 95].

*The Sensory Experiences Questionnaire Short Form (SEQ-SF* *[*96*];) Version 2.1 i*s a 41-item caregiver report questionnaire that measures behavioral responses to common everyday sensory experiences in young children with autism and other developmental disabilities from 6 months to 6 years old. An adapted version of the SEQ-SF (for infants aged 3–5 months old) will be used at Time 1. The SEQ-SF measures sensory hyporeactivity, sensory hyperreactivity and sensory seeking behaviors across different sensory modalities and contexts. The SEQ-SF has been shown to exhibit good reliability and validity in typically developing children as well as children with autism and other developmental delays [96–99].

*Brief Infant Sleep Questionnaire – Revised Short Form (BISQ-R SF* *[*100*];)* is a caregiver report questionnaire that measures infant and toddler sleep problems, validated for ages 0 to 29 months. The questionnaire includes three subscales: infant sleep, parent perception, and parent behaviour. The BISQ-R SF has been shown to exhibit high test-retest reliability and validity [100, 101].

#### Everyday executive functioning

*The Early Executive Functions Questionnaire (EEFQ* *[*102*];)* is a 31-item caregiver report questionnaire that assesses impairments in everyday EF, developed for infants and toddlers aged 9 to 30 months old [102]. It is comprised of items measuring inhibitory control, flexibility, and working memory that load on to a common Cognitive Executive Functioning factor. The EEFQ has demonstrated good internal consistency and convergent validity [102].

#### Early autism behaviour

*The Baby and Infant Screen for Children with aUtIsm Traits Part 1 (BISCUIT-Part 1* [103];) is a 62-item caregiver-report questionnaire used to measure the traits and behaviour associated with autism. The measure has been validated for toddlers between the ages of 17 to 37 months old. The BISCUIT-Part 1 has demonstrated both excellent validity and reliability and has been tested with children with various medical complications, including CP [103–105].

#### Everyday Social and Executive Functioning, and Child Wellbeing at 24 months

Everyday social and executive functioning and child wellbeing will be assessed at 24 months (T4) by administering the following questionnaires to caregivers at the final assessment timepoint.

#### The Vineland Adaptive Behaviour Scales – Third Edition (Vineland-3 [106];)

is a gold-standard measure of adaptive functioning. It has been validated for use in individuals from birth to 90 years of age. The Vineland-3 has robust psychometric properties, with strong internal consistency, test-retest reliability, and inter-rater reliability. It has four domains: communication, daily living skills, socialization, motor skills, and an optional fifth domain for maladaptive behaviour. The Vineland-3 can be administered to the primary caregiver in a semi-structured interview format by a research/clinical professional. Alternatively, it can be completed by a caregiver as a rating form. The Vineland-3 has been validated in children with a range of developmental conditions, including autism [107], and has been used in children with CP [108].

*The Behavior Rating Inventory of Executive Functioning – Preschool Version (BRIEF-P* *[*109*];)* is a 63-item parent report questionnaire that assesses impairments in everyday executive functioning in children 2 to 5 years old. It has two broad indexes: behavioral regulation and metacognition, as well sub-scale scores measuring inhibition, shifting, emotional control, working memory, and planning/organization. The BRIEF-P has been validated in typically developing children, as well as children with learning, neurological, and developmental conditions [110].

*The Child Behavior Checklist for Ages 1.5–5 (CBCL* *[*111*];)* is widely used to assess emotional and behavioural disorders in preschool-aged children 1.5 to 5 years. It has demonstrated validity and reliability based on an independent factor analysis in children with autism [112], and has been used with children with CP [113, 114].

#### Autism traits and behaviour

##### Early autism traits and behaviour

Early autistic behaviour will be assessed at 8–12 (T2) and 18 (T3) months using the Autism Observation Scale for Infants (AOSI) – a semi-structured observational assessment designed to study the nature and emergence of autism-related behavioural markers in infants from 6 to 18 months old [115]. The AOSI is designed to prompt and observe behaviours considered to be early behavioural indicators of autism (e.g., imitation, orientation to name, social interest and shared affect, atypical sensory behaviour, and eye contact). The AOSI has been used in infants at risk for autism [35] and has demonstrated strong psychometric properties [115].

##### Comprehensive assessment of autism at 24 months

A comprehensive assessment of autism will be administered to children at 24 months (T4) using the ADOS-2 toddler module [116]. The ADOS-2 is a standardised semi-structured diagnostic tool that measures autism traits and behaviours. This task is comprised of play-based activities and questions designed to prompt and observe the communicative, social, and stereotyped behaviours which are relevant to the diagnosis of autism. Observation of behaviour will be coded according to the ADOS-2. The ADOS-2 has strong inter-rater and test-retest reliability for individual items, strong inter-rater reliability within domains and excellent internal consistency [117, 118].

#### Social attention

An eye tracking task will be administered to measure social attention at 24 months (T4). In this task, the infant will watch a video of a shared book reading scenario, which is incorporated with multiple bids for joint attention. Social attention will be measured by tracking eye gaze to the social and non-social stimuli throughout the video and during the joint attention episodes. Eye-tracking data will be collected using an integrated TX300 eye tracker with a sampling rate of 300 Hz and equivalent gaze accuracy at 0.4 degrees (Tobii Technology, Stockholm, Sweden). This shared book reading eye tracking task has demonstrated utility in detecting atypical social attention in autistic children aged 3 to 12 years old [119].

#### Other assessments

Select assessments measuring motor, cognitive and language development, caregiver well-being and caregiver perceived stigma will be used to explore interactions with markers of social and executive impairment. Some of these assessments are collected as part of standard clinical practice across our participating CPA early diagnosis clinics and NICU follow-up clinics. Where possible, data for these assessments will be shared with the research team, rather than repeating the assessments. If assessments have not been completed or we cannot access these data, the following assessments will be administered by the researchers during the assessment visits.

##### Motor assessments

The Gross Motor Function Measure (GMFM-66 [120]; specific to infants with, or a risk for, CP) and the Peabody Developmental Motor Scales-2 (PDMS-2 [121];) will be used to assess early gross and fine motor function, respectively.

##### Language and cognitive development assessments

Language and cognitive development will be assessed using the Bayley Scales of Infant and Toddler Development-4 (Bayley-4 [122];). The Bayley-4 includes cognitive, language, motor, social-emotional and adaptive behaviour scales.

##### Caregiver well-being

Caregiver well-being will be measured using the Depression Anxiety Stress Scale (DASS-21 [123];). The DASS-21 is a self-report measure assessing the frequency and severity of negative emotions. Caregiver perception of community stigma around developmental delay will be measured using a variation of the ASD stigma questionnaire [124]. This stigma questionnaire is a self-report measure that assesses caregivers’ perception of stigma around developmental delay in their communities.

### Analytic plan

#### Sample size justification

Allowing for 10% attrition, and based on estimated effect sizes from prior studies (which revealed moderate to large effects when examining early markers of social and executive functioning, and risk for autism [125–127];), a sample size of *N* = 90 will yield power of 0.97 (f^2^ = 0.30, α = 0.01). While this sample size is larger than that suggested by an a priori power analysis (*N* = 71, based f^2^ = 0.30, α = 0.01, 1-ß = 0.90), this will enable additional exploratory analyses to determine broader social-emotional, general developmental and epidermal predictors of social and executive function as well as the development of autism.

#### Data analysis

Multilevel modeling will evaluate the relationship between behavioural and neurobiological measures from our social and executive function assessments at Times 1–3 (e.g., amount of eye gaze and facial mimicry on social tasks, and percent accuracy on executive functioning tasks) and the social function and autism outcomes (as assessed by the ADOS-2 and social attention task) as well as the executive function outcome (as assessed by the BRIEF-P) at 24 months (T4). Multilevel models are appropriate for handling missing data and provide an unbiased estimate of the means at each time point while retaining the total sample. A multiple regression will assess the relationship between early social development and executive functioning measures and motor, language and child well-being and everyday functioning assessments. Multiple imputation strategies will be employed to deal with any missing data in regression analyses. Imputed data will not be incorporated into any raw or primary datasets. The priority will be to minimize missing data, and the study will have a protocol for follow-up that maximizes data in all participants. Finally, we will conduct exploratory analysis on subgroups of infants with different conditions (e.g., CP, congenital heart disease, prematurity) and combined comorbidities to understand how social and executive function delays may differ across these subgroups.

## Discussion

This is the first study, we are aware of, to prospectively track the development of social and executive function as well as the risk for autism in CP and NICU graduates from early infancy through to 2 years of age. The study will take a multi-modal approach, integrating behavioural, neurobiological, and caregiver-rated everyday functioning markers, to identify the earliest signs of developmental delay. This will constitute a critical step towards the earliest detection and intervention opportunities in these high-risk cohorts. The findings of this study will also expand our knowledge on the early emergence of autism across the wider spectrum, beyond infants at familial risk for autism [43, 44]. The increased autism prevalence estimates in CP (up to 30% [32];) compared to the rate found in high-risk siblings (3–18% [128];) highlights a key advantage of investigating early markers of autism in this population. Similarly, given that prematurity, low birth weight and other neonatal medical complications (e.g., congenital heart disease) that warrant admission to the NICU have been identified as key risk factors for autism [26–31, 33, 129–132], this study holds the potential to deepen our understanding of early markers of autism in a more diverse, high-risk cohort. This broader understanding of autism will be pivotal to the development of more personalised intervention and supports.

One potential limitation of this study is the clinical heterogeneity of the study sample, which includes infants with, or at risk for, CP as well as NICU graduates, who are likely to present with various medical complications. This will be addressed by conducting exploratory subgroup analyses to examine how delayed developmental trajectories change across infants with different clinical presentations (e.g., CP, congenital heart disease, prematurity) and combined comorbidities. An inherent challenge to longitudinal studies is the potential for poor retention and follow-up. This study has been designed to minimize attrition rates by limiting the number of assessment visits, and by streamlining the questionnaires into a single online portal, with the option of completing the questionnaires at home. In addition to this, the sample size has been set to allow for 10% attrition. Another methodological challenge for this study is the limited availability of tools validated for infants with CP, who are likely to present with sensory and motor impairments. In consideration of this, some assessment tools have been modified to ensure that the motor problems do not interfere with the task performance (e.g., looking version of the A-not-B task [79, 80];) and measures tested with children with CP have been selected when available (e.g., BISCUIT-Part 1 [104];).

The results of this study will allow us to identify the potential behavioural, neurobiological, and everyday functioning markers of social and executive function delays in CP and NICU graduates at the earliest point in time. Furthermore, it will provide insights into early markers which may increase risk for autism in infants with CP and NICU graduates. To date, we have very little understanding of whether early social and executive functioning markers can be used to track developmental divergence over time in high-risk infants, and if they can predict risk for autism. Therefore, the results of this study will provide critical knowledge on early detection of delays in high-risk infants. This will ultimately inform the development of better, timely, and targeted detection and intervention approaches to optimize developmental outcomes in these high-risk cohorts.

## Data Availability

No datasets were generated or analysed during the current study.

## Abbreviations

ADOS-2: Autism diagnostic observation schedule, 2nd edition
AOSI: Autism observation scale for infants
ASQ-3: Ages and stages questionnaire, third edition
ASQ:SE-2: Ages and stages questionnaire: social-emotional, second edition
Bayley-4: Bayley scales of infant and toddler development-4
BISCUIT-Part 1: Baby and infant screen for children with autism traits part 1
BISQ – R SF: Brief infant sleep questionnaire – revised (short form)
BITSEA: Brief infant-toddler social and emotional screening
BRIEF-P: Behavior rating inventory of executive functioning – preschool version
CBCL: Child behavior checklist for ages 1.5–5
CP: Cerebral palsy
CPA: Cerebral palsy alliance
CSBS-DP ITC: Communication and symbolic behavior scales developmental profile infant-toddler checklist
DASS-21: Depression anxiety stress scale
ECBQ-SF: Early childhood behavior questionnaire – short form
EEFQ: Early executive functions questionnaire
EEG: Electroencephalogram
GMFM: Gross motor functioning measure;
IBQ-r SF: Infant behavioral questionnaire-revised short form
ICEP: Infant and caregiver engagement phases
MACI: Manchester assessment of caregiver-infant interaction
NICU: Neonatal intensive care unit
NNNS: NICU network neurobehavioural scale
PDMS-2: Peabody developmental motor scales-2
SEQ-SF: Sensory experiences questionnaire short form
Vineland-3: Vineland adaptive behaviour scales – third edition
